# Draft Genome Sequence of *Sphingobium* sp. Strain BHC-A, Revealing Genes for the Degradation of Hexachlorocyclohexane

**DOI:** 10.1128/genomeA.00254-14

**Published:** 2014-04-03

**Authors:** Chao Xue, Li Cao, Rong Zhang, Jian He, Shunpeng Li, Qing Hong

**Affiliations:** a Key Laboratory of Agricultural Environmental Microbiology, Ministry of Agriculture, College of Life Sciences, Nanjing Agricultural University, Nanjing, China; b National Engineering Research Center for Organic-Based Fertilizers and Jiangsu Collaborative Innovation Center for Solid Organic Waste Utilization, College of Resources and Environmental Sciences, Nanjing, China

## Abstract

Here, we report the draft genome sequence of *Sphingobium* sp. strain BHC-A, a *lin* gene-based hexachlorocyclohexane (HCH)-degrading strain, isolated from soil that suffered long-term HCH contamination in an insecticide factory.

## GENOME ANNOUNCEMENT

Technical hexachlorocyclohexane (t-HCH) mainly consists of α-, β-, γ-, and δ-isomers (1, 2). Only γ-HCH has insecticidal activity. Both t-HCH and γ-HCH were used worldwide between the 1940s and the 1990s to control a wide range of agricultural, horticultural, and public health pests. Now, they are banned or restricted in most countries because of their toxicity and persistence in the environment. However, these insecticides are still being manufactured and used as a cheap but effective insecticide in some developing countries, mainly for economic reasons (3). Therefore, residue problems of HCH will continue for a long time. Bioremediation has become a viable and promising biotechnological approach to cleaning up polluted environments. An aerobic bacterium, *Sphingobium* sp. strain BHC-A, was isolated from soil that suffered long-term HCH contamination in an insecticide factory (4). This strain degraded α-, β-, γ-, and δ-HCH. The *linABCDEFR* genes have been cloned, and they all show high identity to the counterparts of *Sphingobium japonicum* UT26, except *linB*. The LinB proteins of strain BHC-A have seven heterogeneous amino acid residues in sites similar to those of LinB from UT26, which determine the special role of LinB in the transformation of β- and δ-HCH (5, 6). To further investigate the degradation pathways of β- and δ-HCH as well as other recalcitrant xenobiotics, we sequenced the genome of strain BHC-A.

Genome DNA was extracted using the Qiagen Genomic-tip 20/G kit, following the manufacturer’s instructions. Paired-end genome sequencing was performed for a 350-bp DNA fragment on an Illumina Solexa sequencing platform at the Beijing Genome Institute, Shenzhen, China. In total, 632.64 Mb of raw data was obtained and 500 Mb of data passed quality trimming. The genome was assembled by Velvet (v 1.2.10) (7) with a 31-base k-mer, resulting in 217 scaffolds larger than 500 bp. The maximum length of these scaffolds was 132,651 bp. The total length of the genome was estimated to be 4,063,496 bp, with a GC content of 65.3%. Genome annotation was performed using the RAST server with the SEED database (8) and the NCBI Prokaryotic Genomes Automatic Annotation Pipeline (http://www.ncbi.nlm.nih.gov/genomes/static/Pipeline.html).

The chromosome of the strain harbors 3,537 protein-coding sequences, 391 subsystems, 45 tRNAs, and 2 rRNAs. Fourteen *lin* genes, including *linBCDEFGHIJKLMNR*, were annotated and located in the draft genome. However, *linA* was not found, and we speculate that *linA* is in the gaps, because its length is only 471 bp and our previous study also proved the existence of *linA* (5). Data and information from this study will be used and a comparative analysis with other genomes of HCH-degrading sphingomonads (9–17) will be carried out to increase understanding of HCH degradation, especially the evolution and acquisition of *lin* genes in sphingomonads.

### Nucleotide sequence accession numbers.

The draft genome sequence of *Sphingobium* sp. BHC-A has been deposited in GenBank under the accession number JDRU00000000. The version described in this paper is version JDRU01000000.
